# Effects of the Cooling Temperature at the Farm on Milk Maturation and Cheesemaking Process in the Manufacture of Parmigiano Reggiano PDO Cheese

**DOI:** 10.3390/ani11102835

**Published:** 2021-09-28

**Authors:** Piero Franceschi, Milena Brasca, Massimo Malacarne, Paolo Formaggioni, Michele Faccia, Giuseppe Natrella, Andrea Summer

**Affiliations:** 1 Department of Veterinary Science, University of Parma, Via del Taglio 10, I-43126 Parma, Italy; piero.franceschi@unipr.it (P.F.); andrea.summer@unipr.it (A.S.); 2 Institute of Sciences of Food Production, National Research Council, Via Celoria 2, IT-20133 Milan, Italy; milena.brasca@ispa.cnr.it; 3 Department of Soil, Plant and Food Sciences, University of Bari, Via Amendola 165/A, 70125 Bari, Italy; michele.faccia@uniba.it (M.F.); giuseppe.natrella@uniba.it (G.N.)

**Keywords:** Parmigiano Reggiano cheese, milk cooling at the farm, milk dairy maturation, cheesemaking efficiency, milk salt equilibria, milk microbiological characteristics

## Abstract

**Simple Summary:**

Currently, the official protocol of Parmigiano Reggiano, a hard, cooked Italian PDO cheese, prohibits cooling the milk at the farm below 18 °C. As Parmigiano is made from raw milk, the microbiological characteristics of the raw matter have a strong impact both on the cheesemaking process and cheese quality. A lower cooling temperature, i.e., 9 °C, could better keep under control the microbial growth rate, which affects both the total bacterial count and specific species that are detrimental for dairy products. In this research, the impact of keeping milk at the farm at 9 °C on milk quality and cheese characteristics was investigated. The outcomes of the study showed that while the microbiological aspects benefited from the lower temperature, the coagulation properties of milk tended to worsen and caused slightly higher losses of macroconstituents in the whey. Nevertheless, the cheesemaking process and cheese features were not substantially modified, suggesting that the improvement of the microbiological aspects largely offsets the worsening of coagulation properties. The results of this research represent a scientific contribution to get to a formal request for modification of the Parmigiano Reggiano regulation, which, once accepted, could improve the hygienic characteristics of milk and, possibly, the final quality of Parmigiano Reggiano cheese.

**Abstract:**

Parmigiano Reggiano is a hard PDO cheese made from bovine raw milk, whose microbiological characteristics have important repercussions on cheese quality. According to the EU official production protocol, milk temperature at the farm must not drop below 18 °C. The present research aimed to study the effect of cooling milk at the farm at 9 °C on the characteristics of milk and on the cheesemaking process and losses during manufacture. Six cheesemaking trials were performed in two different dairies. In each of them, two cheesemakings were made in parallel: one with milk kept at 9 °C (TM9) and the other with milk kept at 20 °C (TM20). TM9 milk, in comparison with TM20, showed after the creaming process a significant reduction not only of total bacterial count but also of psychrotrophic and lipolytic bacteria. At the same time, TM9 milk showed a higher creaming capacity and, consequently, a lower fat content than TM20. TM9 vat milk had worst coagulation properties than TM20, which caused slightly higher loss of fat and curd fines into the whey. Nevertheless, these changes were too small to influence the efficiency of the cheesemaking process; conversely, maintaining milk at the farm at 9 °C led to a reduction of the number of spoilage bacteria.

## 1. Introduction

Parmigiano Reggiano (PR) is an Italian hard cooked, Protected Designation of Origin (PDO) cheese made from raw bovine milk following a strict manufacturing protocol [1]. It is ripened for a period that can extend over 24 months (minimum 12 months) and, as for all other raw milk cheeses, the chemical composition and the hygienic characteristics of the raw matter have important repercussions on the yield and quality of the final product [2,3].

The total bacterial and somatic cell counts are the most important hygienic parameters, and an increase of their value is generally associated with worsening of the milk cheesemaking properties [4], with negative repercussion on the milk cheese yielding ability [5]. In particular, the increase of the somatic cell count deeply worsens the rennet coagulation properties, resulting in a curd with reduced capacity of retaining the fat globules in the para-casein matrix [6,7], and in increased cheesemaking losses due to the high level of fat lost into the whey [6].

In addition to this, the increase of somatic cells in milk is associated to an increase of the activity of the proteolytic enzymes, mainly plasmin, which causes an early degradation of caseins into fragments of different size [8,9].

On the other hand, it is known that an increased bacterial count can lead to the risk of abnormal fermentations that can result in more or less pronounced defects of the cheese texture, colour, and flavour [10].

In the PR cheesemaking process, the level of both somatic cell and bacterial counts is influenced by the spontaneous creaming that takes place overnight at room temperature, since these cells tend to adhere to the membrane of the fat globules. This operation is pivotal to decrease the number of clostridia spores that are responsible for late blowing during cheese ripening. Moreover, spontaneous defatting determines substantial changes in the chemical and microbiological composition of milk, whose extent depend on its initial characteristics, with potential depletion of spoilage bacteria and enrichment in lactic acid bacteria [11,12].

The official production protocol of PR cheese requires that the cows are milked two times a day and that the time between the beginning of milking and the delivery of milk to the cheese factory must be shorter than 7 h [1]. Moreover, in this period, the milk may be cooled at the farm and during transport to the cheese factory, but the temperature must not drop below 18 °C [1], since refrigeration at 4 °C might favour the growth of psychotropic bacteria [13,14,15], which produce thermoresistant proteolytic and lipolytic enzymes [14,16] responsible for flavour defects during cheese ripening [17,18]. Furthermore, extended cooling favours the solubilisation of β-casein and inorganic calcium phosphate from the casein micelle, leading to a worsening of the rennet coagulation and cheese-yielding abilities of milk [13,19,20].

However, cooling milk at 9 °C, as is done for other PDO hard cheeses such as Grana Padano, could be a suitable compromise to safeguard the quality of the milk destined to the transformation into PR cheese.

It is known that the modification of any processing parameter in PDO cheeses is not allowed: any variation must be carefully evaluated to verify its compatibility with the desired characteristics of the final product; only after the necessary verifications, a request of modification of the official protocol can be asked to the EU Commission.

In a previous paper, the effect of the cooling temperature of the milk at the farm on PR proteolysis was investigated [21]; thus, the present research aimed to compare the effect of cooling milk at the farm at 9 °C vs. 20 °C on the chemical and microbiological characteristics of the milk, on the cheesemaking process, and on the cheesemaking losses during manufacture.

## 2. Materials and Methods

### 2.1. Experimental Design

The study was carried out in two cheese factories normally engaged in PR cheese production. A total of 6 cheesemaking trials were performed. In each trial, two PR cheesemakings were performed in parallel: one made with milk kept at the farm and transported to cheese factory at 9 °C (TM9) and the other with milk kept and transported at 20 °C (TM20).

Both TM9 and TM20 were collected from the same farm by the application of a T-shaped tube that conveyed the milk leaving the milk parlour into two tanks kept at the two different temperatures. Milking lasted about 3 h, and the transport to the cheese factory took about 1 h.

### 2.2. Cheesemaking Process

The cheesemaking process was carried out according to the official EU protocol [1] except for the milk-cooling temperature of TM9. Once arrived at the cheese factory, the full cream evening milk was transferred into the creaming tank, where it underwent the overnight natural creaming process at room temperature [11].

The morning after, the partially skimmed evening milk, present in the lower part of the creaming tank, was taken from the bottom of the tank and transferred into the cheesemaking vat, where it was added with the full cream milk obtained during the morning milking (ratio 1:1). The resulting mixture—about 1200 kg of milk called “vat milk”—had a fat content of about 2.6% and a fat to casein ratio of about 1:1. A natural whey starter was inoculated, which was obtained by the spontaneous acidification of the whey of the previous day cheesemaking. Then, the inoculated vat milk was heated to 33 °C and coagulated with calf rennet (3 g/100 kg of milk, strength, 1:120,000, Chr. Hansen, DK-2970 Hørsholm, Denmark). Coagulation occurred in about 10 min, and the curd was then cut into small granules, of approximately 4 mm of diameter, and “cooked” by raising the temperature up to about 55 °C. After cooking, the small curd particles were left to settle spontaneously on the bottom of the vat for about 1 h to form a cheese mass. During this time, the temperature remained between 50 and 55 °C. Then, the cheese mass was extracted from the vat and divided into two parts, which were placed into the moulds for two days to form two cheese wheels. After that, the two wheels were salted in saturated brine (about 370 g/L NaCl), for a period of 18–20 days. Then, they entered the ripening room where they remained for 21 months.

The wheels produced with TM9 milk were not branded as PDO Parmigiano Reggiano.

### 2.3. Sampling Procedure and Classification of Batches

For each cheesemaking trial, the following samples were collected:

– Full cream evening milk (FC-milk) was taken directly from the creaming tank before starting the creaming process;
– Partially skimmed milk (PS-milk) was taken at the end of the creaming process;
– Vat milk (V-milk) was collected directly from the cheesemaking vat, after the commingling of the PS-milk and the full cream milk (5 min of stirring) and before the addition of the natural whey starter;
– Cooked whey (C-whey), which is the whey that remains in the vat after the extraction of the curd that was “cooked” at 55 °C; it was collected from the vat after the curd extraction.

### 2.4. Analytical Methods

Total, non-casein, and non-protein nitrogen fractions were determined by Kjeldahl method respectively on milk (FC-milk, PS-milk, and V-milk), on milk acid whey at pH 4.6, and on filtered whey obtained after the addition of 120 g/L trichloroacetic acid (TCA, Carlo Erba Reagents, I-20010 Milan, Italy) [22,23,24]. The Kjeldahl method was also used to quantify the non-coagulable [25] (on rennet whey) and the proteose-peptone [26] nitrogen contents in FC-milk, PS-milk, and V-milk. Rennet whey was obtained according to Verma and Sommers [25]. In short, 2 mL of diluted rennet 1:100 *w*:*v* (Christian Hansen, DK-7172 Hørsholm, Denmark) were added to 100 mL of milk and, after coagulation, the obtained whey was filtered on Whatman 1 paper filter (Merck Millipore Corporation, D-64293, Darmstadt, Germany).

Kjeldahl analyses were always performed using a DK6 Digestor and an UDK126A Distiller (VELP Scientifica, I-20865, Usmate, Italy); from the obtained nitrogen data, crude protein, casein, NPN × 6.38 and proteose-peptones were calculated as described by Malacarne et al. [11], and non-coagulable protein was calculated according to Franceschi et al. [27].

Crude protein and casein were determined, also on C-whey, using the same method used for their determination on milk.

Lactose on FC-milk, PS-milk, and V-milk and fat content on FC-milk, PS-milk, V-milk, and C-whey were determined by mid-infrared method [28] using a MilkoScan FT 6000, (Foss Electric, DK-3400, Hillerød, Denmark). Dry matter and ash content on V-milk were obtained after oven-drying milk at 102 °C [29] and after muffle calcination at 530 °C [30], respectively. From ash dissolved in hydrochloric acid (2N; Carlo Erba Reagents, I-20010 Milan, Italy), as described by Malacarne et al. [31], the total contents of calcium and magnesium were determined by Atomic Absorption Spectrometry (Perkin-Elmer 1100 B, Waltham, MA 2451, USA), whereas the total content of phosphorus was determined, always on chloridric ash solution, but by the colorimetric method [31]. In short, 10 mL of hydrochloric ash solution diluted 40 times were placed in a test tube with 2 mL of perchloric acid, 2 mL of a 2% (*p*/*v*) solution of 2:4 diamino-phenol-hydrochloride, and 20% (*p*/*v*) of sodium metabisulfite and 1 mL of 8.3% (*p*/*v*) solution of ammonium molybdate (Carlo Erba Reagents, I–20010 Milan, Italy). After 25 min, 1 mL of this solution was read at 750 nm with a spectrophotometer Helios (Thermo Fisher Scientific, Waltham, MA, USA).

Moreover, each V-milk sample, previously skimmed, was also submitted to an ultrafiltration process in Amicon 8200 ultrafiltration cells (Merck Millipore Corporation, Darmstadt, Germany) as described by Malacarne et al. [32], and from this hydrochloric ash were determined the milk’s soluble fractions of calcium, magnesium, and phosphorus with the same methods used for the determination of their total contents. From these values, the contents of colloidal Ca, P, and Mg were calculated as follows [33]:

CMC = TMC − SMC,(1)

where CMC is the colloidal content, TMC is the total content, and SMC is the soluble content (all three expressed in mg/100 g).

Furthermore, colloidal P content was corrected for the quota of P of phospholipids, always according to Malacarne et al. [33].

Titratable acidity and pH values were measured on FC-milk, PS-milk, and V-milk samples by potentiometer and titration of 50 mL of milk with 0.25 N sodium hydroxide with Soxhlet-Henkel method [34], respectively, both by Crison Compact Titrator D (Crison Instruments, E-08328 Barcelona, Spain).

The rennet coagulation parameters, clotting time (r), curd-firming time (k_20_), and curd firmness (a_30_) were measured at 35 °C on FC-milk, PS-milk, and V-milk (McMahon and Brown 1982) using Formagraph (Foss Electric, DK-3400, Hillerød, Denmark). In short, the method provides that 0.2 mL of a diluted rennet solution (final title 1:19000; Chr. Hansen, I-20094 Corsico MI, Milan, Italy) are added to 10 mL of milk, and that right after, the sample is inserted into the Formagraph device.

Somatic cells and total bacterial count were determined on FC-milk, PS-milk, and V-milk using the fluoro-opto-electronic method [35] with Fossomatic (Foss Electric, Hillerød, Denmark), and using the flow cytometry method [36] with BactoScan FC (Foss Electric, Hillerød, Denmark), respectively.

For microbiological analyses, serial decimal dilutions were prepared and plated on the following media: De Man, Rogosa, Sharpe Agar (MRS, Oxoid) (21 °C for 5 days) to count mesophilic lactic acid bacteria [37]; Plate Count Agar (PCA, Oxoid) (6.5 °C for 10 days) for the enumeration of psychrotrophic bacteria [38]; Litmus milk (BD Diagnostics, Sparks, MD, USA) (30 °C for 48 h) to count proteolytic bacteria; Tributyrin agar (Oxoid) at 30 °C for 7 days for lipolytic bacteria [39]. For statistical analysis, microbial counts were transformed into logarithms of colony-forming units per mL (Log CFU/mL).

Curd fines were measured on each C-whey sample as described by Franceschi et al. [40]. Briefly, C-whey was centrifuged, and the pellet transferred on a Whatman 40 filter paper; then, it was dried at 102 °C and weighed.

From the value of fat and casein, the fat to casein ratio was calculated, and from the fat value of FC-milk and PS-milk, the creaming capacity was calculated as follows:

Creaming capacity (%) = (fat FC-milk − fat PS-milk) × 100/fat FC-milk,(2)

where fat FC-milk = fat in full cream milk (%), and fat PS-milk = fat in partially skimmed milk (%).

Finally, the estimated cheesemaking losses (ECL) values of protein, casein, and fat were calculated according to Franceschi et al. [41] as follows:

ECL = (M-whey) × 100/(V-milk),(3)

where ECL is expressed as percentage; (M-whey) = concentration in whey, expressed as g/100; (V-milk) = concentration in milk, expressed as g/100 g.

### 2.5. Statistical Analysis

Statistical analysis for the comparison between FC-milk and PS-milk was performed by ANOVA using the SPSS (IBM SPSS Statistics vers. 27, Armonk, NY, USA) procedure with the following general linear model:

Y_ijk_ = µ + M_i_ + T_j_ + ε_ijk_, (4)

where Y_ijk_ = dependent variable; µ = overall mean; M_i_ = effect of the milk type, FC-milk or PS-milk (i = 1, 2); T_j_ =effect of trial (j = 1, ..., 6); ε_ijk_= residual error.

Moreover, statistical analysis for the comparison between TM9 and TM20 at the farm was performed by ANOVA using always the SPSS procedure with the following general linear model:

Y_ijk_ = µ + C_i_ + T_j_ + ε_ijk_, (5)

where Y_ijk_ = dependent variable; µ = overall mean; C_i_ = effect of the temperature, 9 °C or 20 °C (i = 1, 2); T_j_ =effect of trial (j = 1, ..., 6); ε_ijk_= residual error.

Finally, for both models, the significance of the differences was tested always by means of Bonferroni method.

## 3. Results and Discussion

### 3.1. Effect of the Temperature on the Milk Maturation Process

Table 1 shows the chemical composition, physicochemical properties, and microbiological quality of FC-milk and PS-milk at 9 °C or 20 °C.

The main parameters of FC-milk kept at both temperatures fulfilled the requirements for the production of Parmigiano Reggiano cheese. Their values matched with those obtained by Summer et al. [2] in research conducted on 504 bulk milk samples collected monthly in 14 farms over 3 years (average values of 3.62 g/100 g for fat, 3.30 g/100 g for protein, and 3.27 °SH/50 mL for titratable acidity). The TBC and SCC values agreed with those reported by Franceschi et al. [4] in a research study conducted on 1080 bulk milk samples (average values of TBC 45,000–95,000 CFU/mL, SCC 209,000–382,000 cells/mL). PS-milks obtained from both TM9 and TM20 milk showed higher non-coagulable protein and proteose-peptone contents and lower fat content compared to the corresponding FC milk. This is in agreement with the results of Malacarne et al. [11], who reported an increase of proteose-peptones (from 13.22 to 19.07 g/100 g) during the natural creaming process. It should derive from a partial breakdown of casein by plasmin [42] and by proteases of microbial origin [43], as confirmed by the increase of non-coagulable protein.

Regarding the milk kept at 20 °C, lactose, crude protein, casein, non-coagulable protein, and somatic cell content differed significantly between FC-milk and PS-milk. At this temperature, total bacterial count increased significantly during natural milk creaming (*p* ≤ 0.05), while no difference was evidenced for psychrotrophic and lipolytic bacteria.

**Figure 1:**
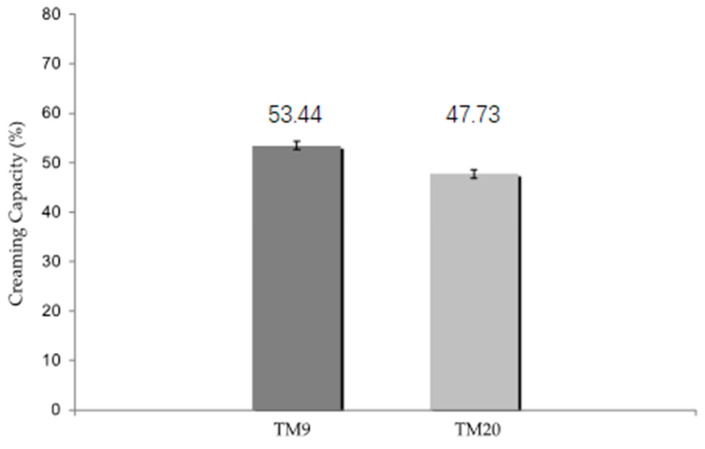
Least square mean values of creaming capacity of the partially skimmed milk (PS-milk) obtained from bulk milk kept at the farm at 9 °C (TM9) and at 20 °C (TM20), TM9 and TM20 means values differ with a *p*-value ≤ 0.05.

Instead, when milk was kept at 9 °C, no significant differences between FC-milk and PS-milk were found for crude protein and casein, whereas a significant reduction was provided by milk creaming for total bacterial count (*p* ≤ 0.05) and for psychrotrophic (*p* ≤ 0.05) and lipolytic bacteria (*p* ≤ 0.01). It is worth noting that the content of mesophilic lactic acid bacteria was not significantly reduced. At both temperature conditions, the content of clostridia spores was reduced, and fat and somatic cell content showed a very significant reduction (*p* ≤ 0.001) as a result of the creaming process, while proteose-peptones significantly increased (*p* ≤ 0.001) probably as a result of the activity of endogenous milk proteases. However, significant differences were observed comparing PS-milk obtained through natural creaming at 9 °C and 20 °C. Milk kept at the farm at 9 °C showed a higher creaming capacity (Figure 1) and, consequently, a lower fat content compared to the milk kept at the farm at 20 °C. For milk kept for 12 h at 8–10 °C, compared to 13–15 °C, Malacarne et al. [11] reported a significant difference of the fat content (3.62 g/100 g vs. 2.07 g/100 g, respectively) and Malacarne et al. [20] observed a higher creaming capacity (42 vs. 38.5%, respectively).

The higher fat content reduction in the milk cooled at 9 °C provided a greater decrease of the somatic cell content in the corresponding PS-milk. It is known that during the creaming process, the somatic cells tend to adhere at the surface of the rising fat globules and, consequently, they remain entrapped into the cream [44]. The reduction of the somatic cell count observed in both milk samples, which was irrespective of the temperature at which the milk was kept before the creaming process (for FC-milk 264,667 vs. 274,500 cells/mL; *p* > 0.05), agreed with a previous study that reported an average value of somatic cells in the partially skimmed milk of 19,000 cells/mL [11]. It is important to consider that the somatic cell content in milk is negatively correlated to the cheese yield in the production of Parmigiano Reggiano cheese [5,7], as well as for other cheeses such as cheddar [19] and cottage [45]. However, it should be considered that the reduction of cheese yield is not directly due to the somatic cells but is the result of modification of milk chemical composition induced by the mammary gland inflammation process [8,46]. The mammary gland inflammation, indeed, is responsible for the decrease of milk casein content [47] that, together with the milk fat content, is the parameter that most influences the cheese yield in Parmigiano Reggiano production [3]. Somatic cells are mainly constituted by leucocytes that have, inside their lysosomes, a lot of enzymes with non-specific lithic functions [9], which can negatively influence the ripening process of the cheese. In the present study, although significant, the difference between the somatic cell content of the PS-milk obtained from the milk previously kept at 9 °C and to the one held at 20 °C was too low (6500 cells/mL) to negatively affect the cheesemaking process and the cheese ripening.

A greater difference was detected in TBC of PS-milk obtained from the milk kept at 9 °C with respect to the one obtained at 20 °C (13,917 vs. 92,666 CFU/mL, *p* ≤ 0.05). Such a difference was the result of the combined effect of the higher creaming capacity and better control of the growth of mesophilic bacteria during the milk maturation process. These results confirm the data reported by Malacarne et al. [11,20], showing a significant difference of the TBC value between the milk kept at 8–10°C for 12 h and the milk kept at 13–15 °C (67,600–100,000 CFU/mL vs. 104,700–218,800 CFU/mL, respectively).

PS-milk obtained from the milk kept at the farm at 9 °C was characterised by a lower content of mesophilic lactic acid bacteria as well as psychrotrophic and lipolytic bacteria.

### 3.2. Effect of the Temperature on the Cheesemaking Process

In Table 2, the chemical composition, physicochemical properties, and microbiological characteristics of the vat milk obtained from milk kept at 9 °C and 20 °C are shown.

Moreover, the estimated cheesemaking losses (ECL) of vat milk obtained from the milk kept at 9 °C and at 20 °C are shown in Table 3.

The fat and dry matter contents and the fat to casein ratio value resulted lower in V-milk obtained by milk kept at 9 °C compared to the one kept at 20 °C (*p* < 0.05). This is due to the higher creaming capacity and consequently lower fat content of the PS-milk obtained from the first milk compared to the second one. This observation is confirmed by the similar values of matter (not fat content) of V-milk derived from milk kept at 9 °C and that derived from milk kept at 20 °C. Moreover, it is important to note that the difference between the mean values of V-milk fat to casein ratio, although significant, is small, and both average values fall within the range from 1 to 1.1, which is considered optimal in the production of the Parmigiano Reggiano cheese.

These findings are in accordance with the worst creaming capacity for milk kept for 12 h at 13–15 °C compared to the one kept at 8–10 °C reported by Malacarne et al. [20].

The higher creaming capacity of the milk at 9 °C, with respect to 20 °C, is due to the crystallisation of fat globules and their breaking, with consequent leaking of triglycerides that favours their aggregation in large clusters that tend to surface more rapidly [48].

Moreover, V-milk obtained from T9-milk was characterised by a lower content of proteose-peptones and a little higher content of non-coagulable protein than T20. This latter result was probably determined by the higher dissociation of β-casein from the casein micelle that is favoured at low temperatures [20,49].

V-milk obtained from milk kept at the farm at 9 °C was also characterised by a longer clotting and curd firming time, and lower curd firmness (*p* < 0.05). Moreover, it also showed a higher fat loss and a higher level of curd fines remaining in the vat after the extraction of the cheese wheels. These results agree with the observation reported by Malacarne et al. [20], who highlighted a worsening of the coagulation properties when milk was kept at 8–10 °C compared to keeping at 13–15 °C for 12 h, with a higher clotting time (23.21 vs. 22.14 min) and a lower curd firmness (11.11 vs. 13.41 mm).

The higher losses of fat and curd fines for V-milk at 9 °C was due to the positive correlation between cheesemaking losses and curd firming time and, partially, to the negative correlation between cheesemaking losses and curd firmness [6]. The better coagulation properties of V-milk at 20 °C can explain the lower loss of fat and curd fines. In fact, better coagulation properties can lead to a reduction of losses in the whey, as evidenced by Franceschi et al. [6]. Milk with better coagulation properties (i.e., curd-firming time and curd firmness), in fact, produces a more elastic and resistant curd that better withstand the technological treatments.

## 4. Conclusions

Overall, storing milk at the farm at 9 °C, instead of 20 °C, significantly affected the chemical composition and the technological properties of the milk for Parmigiano Reggiano cheesemaking. The most relevant influence regarded the milk maturation phenomena occurring during the creaming process, which, in turn, affected both the chemical composition and the technological properties of the partially skimmed milk and of the vat milk.

The vat milk obtained from the milk kept at the farm at 9 °C showed a worsening of the rennet coagulation properties and an increase of the content of non-coagulable protein. However, the changes were very small and did not substantially influence the efficiency of the cheesemaking process, as demonstrated by the small differences found in the cheesemaking losses parameters.

The two temperatures applied were equally effective in reducing clostridial spore content following the natural creaming process. Moreover, keeping the milk at 9 °C led to a higher reduction of the total bacterial count, which was mainly due to a reduction of the numbers of psychrotrophic and lipolytic bacteria, both in the partially skimmed milk and in the vat milk. Finally, maintaining milk at the farm at 9 °C caused a reduction of the number of the spoilage bacteria, and this could lead to an improvement of the overall cheese quality.

## Figures and Tables

**Table 1:** Least square mean values of chemical composition, physicochemical properties, and microbiological parameters of full cream milk (FC) and partially skimmed milk (PS) obtained from bulk milk kept at farm at 9 °C (TM9) and at 20 °C (TM20).

		TM9				TM20				
Parameter	Unit	FC Milk	PS Milk	SE ^1^	*p* ^3^	FC Milk	PS Milk	SE ^1^	*p* ^3^	*p* ^4^
		*n*^2^ = 6	*n*^2^ = 6			*n*^2^ = 6	*n*^2^ = 6			
Lactose	g/100 g	4.85	4.92	0.02	*	4.85	4.93	0.02	*	NS
Fat	g/100 g	3.55	1.64	0.09	***	3.51	1.83	0.08	***	*
Crude protein	g/100 g	3.10	3.12	0.02	NS	3.09	3.16	0.02	*	NS
Casein	g/100 g	2.41	2.41	0.03	NS	2.40	2.45	0.01	**	NS
Casein number	%	77.85	77.24	0.44	NS	77.80	77.59	0.29	NS	NS
NPN × 6.38	g/100 g	0.18	0.18	0.01	NS	0.17	0.17	0.01	NS	NS
Proteose-peptones	g/100 g	0.08	0.14	0.01	***	0.09	0.14	0.01	***	NS
Non-coagulable protein	g/100 g	0.87	0.89	0.01	*	0.85	0.89	0.01	*	NS
pH	Units	6.70	6.69	0.01	NS	6.69	6.69	0.01	NS	NS
Titratable acidity	°SH/50 mL	3.24	3.30	0.04	NS	3.28	3.34	0.03	NS	NS
Clotting time (r)	min	19.96	20.17	0.91	NS	18.25	19.75	0.94	NS	NS
Curd firming time (k_20_)	min	5.21	4.54	0.46	NS	4.13	4.50	0.46	NS	NS
Curd firmness (a_30_)	mm	28.73	30.63	2.61	NS	33.84	31.20	2.49	NS	NS
Total bacterial count	CFU/mL	32,667	13,917	6603	*	30,500	92,666	25,771	*	*
Mesophilic lactic acid bacteria	CFU/mL	1917	533	573	NS	1800	5116	2274	NS	*
Psychrotrophic bacteria	CFU/mL	683	200	116	*	1216	1600	328	NS	*
Proteolytic bacteria	CFU/mL	575	300	101	NS	458	1275	470	NS	NS
Lipolytic bacteria	CFU/mL	1350	300	181	**	1850	6200	2849	NS	*
Clostridia spores	Spores/L	44	15	10	NS	19	15	3	NS	NS
Somatic cell count	Cells/mL	264,667	15,333	15,756	***	274,500	21,833	10,637	***	*

^1^ Standard error. ^2^ Number of samples. ^3^ *p*-value of differences between the FC and PS milk: NS *p* > 0.05; * *p* ≤ 0.05; ** *p* ≤ 0.01; *** *p* ≤ 0.001. ^4^ *p*-value of differences between the PS milk of TM9 and PS milk of TM20: NS *p* > 0.05; * *p* ≤ 0.05.

**Table 2:** Least square mean values of chemical composition, physicochemical properties, and microbiological parameters of the vat milk (V-milk) obtained from the bulk milk kept at the farm at 9 °C (TM9) and at 20 °C (TM20).

Parameter	Unit	V-Milk TM9 *n* ^1^ = 6	V-Milk TM20 *n* ^1^ = 6	SE ^2^	*p* ^3^
Lactose	g/100 g	4.88	4.89	0.01	NS
Fat	g/100 g	2.43	2.53	0.03	*
Crude protein	g/100 g	3.09	3.11	0.02	NS
Casein	g/100 g	2.40	2.41	0.02	NS
Casein number	%	77.40	77.53	0.13	NS
NPN × 6.38	g/100 g	0.18	0.17	0.01	NS
Proteose-peptones	g/100 g	0.11	0.12	0.01	*
Non-coagulable protein	g/100 g	0.88	0.86	0.01	**
Dry matter	g/100 g	11.19	11.33	0.04	*
Matter not fat	g/100 g	8.76	8.80	0.02	NS
Fat to casein ratio	Value	1.01	1.05	0.01	*
pH-values	Value	6.69	6.69	0.01	NS
Titratable acidity	°SH/50 mL	3.27	3.28	0.01	NS
Clotting time (r)	min	20.13	19.16	0.18	*
Curd firming time (k_20_)	min	5.21	4.75	0.17	*
Curd firmness (a_30_)	mm	26.92	30.29	1.04	*
Ash	g/100 g	0.70	0.69	0.01	NS
Total Ca	mg/100 g	113.51	112.64	0.91	NS
Colloidal Ca	mg/100 g	73.70	72.62	1.02	NS
Soluble Ca	mg/100 g	39.81	40.03	0.64	NS
Total P	mg/100 g	89.04	88.52	0.26	NS
Colloidal P	mg/100 g	42.75	42.64	0.38	NS
Soluble P	mg/100 g	44.80	44.33	0.29	NS
Total Mg	mg/100 g	10.39	10.18	0.09	NS
Colloidal Mg	mg/100 g	2.75	2.38	0.12	NS
Soluble Mg	mg/100 g	7.81	7.80	0.07	NS
Total bacterial count	CFU/mL	33,167	99,167	24,128	*
Mesophilic lactic acid bacteria	CFU/mL	1167	3333	654	*
Psychrotrophic bacteria	CFU/mL	667	1600	333	*
Proteolytic bacteria	CFU/mL	1058	967	323	NS
Lipolytic bacteria	CFU/mL	1083	6833	3204	*
Clostridia spores	Spores/L	47	28	8	NS
Somatic cell count	Cells/mL	125,667	139,333	6169	NS

^1^ Number of samples. ^2^ Standard error. ^3^ *p*-value: NS *p* > 0.05; * *p* ≤ 0.05; ** *p* ≤ 0.01.

**Table 3:** Least square mean values of estimated cheesemaking losses (ECL) of vat milk (V-milk) obtained from the bulk milk kept at the farm at 9 °C (TM9) and at 20 °C (TM20).

Estimated Cheesemaking Losses (ECL)		V-Milk TM9 *n* ^1^ = 6	V-Milk TM20 *n* ^1^ = 6	SE ^2^	*p* ^3^
Fat	%	14.39	13.53	0.31	*
Protein	%	27.82	27.21	0.20	NS
Casein	%	1.18	0.94	0.16	NS
Curd fines	mg/kg	128.14	98.13	7.75	*

^1^ Number of samples. ^2^ Standard error. ^3^ *p*-value: NS *p* > 0.05; * *p* ≤ 0.05.

## Data Availability

The data presented in this study are available on request from the corresponding author.
